# A Mobile Phone App for the Provision of Personalized Food-Based Information in an Eating-Out Situation: Development and Initial Evaluation

**DOI:** 10.2196/12966

**Published:** 2019-11-04

**Authors:** Katherine Marie Appleton, Jeff Bray, Sarah Price, Gernot Liebchen, Nan Jiang, Ioannis Mavridis, Laure Saulais, Agnès Giboreau, Federico J A Perez-Cueto, Rebecca Coolen, Manfred Ronge, Heather Hartwell

**Affiliations:** 1 Research Centre for Behaviour Change, Department of Psychology Faculty of Science and Technology Bournemouth University Poole United Kingdom; 2 Faculty of Management Bournemouth University Poole United Kingdom; 3 Faculty of Science and Technology Bournemouth University Poole United Kingdom; 4 University of Macedonia Thessaloniki Greece; 5 Food and Hospitality Research Center Institut Paul Bocuse Ecully France; 6 Department of Food Science University of Copenhagen Copenhagen Denmark; 7 Ronge and Partner Baden-bei-Wien Austria

**Keywords:** eating, eating behavior, food, diet, mhealth, mobile app, digitalhealth, smartphone

## Abstract

**Background:**

Increasing pressure from governments, public health bodies, and consumers is driving a need for increased food-based information provision in eating-out situations. Meals eaten outside the home are known to be less healthy than meals eaten at home, and consumers can complain of poor information on the health impact and allergen content of meals eaten out.

**Objective:**

This paper aimed to describe the development and early assessment of a mobile phone app that allows the provision of accurate personalized food-based information while considering individual characteristics (allergies, diet type, and preferences) to enable informed consumer choice when eating out.

**Methods:**

An app was designed and developed to address these requirements using an agile approach. The developed app was then evaluated at 8 public engagement events using the System Usability Scale (SUS) questionnaire and qualitative feedback.

**Results:**

Consideration of the literature and consultation with consumers revealed a need for information provision for consumers in the eating-out situation, including the ability to limit the information provided to that which was personally relevant or interesting. The app was designed to provide information to consumers on the dishes available in a workplace canteen and to allow consumers the freedom to personalize the app and choose the information that they received. Evaluation using the SUS questionnaire revealed positive responses to the app from a range of potential users, and qualitative comments demonstrated broad interest in its use.

**Conclusions:**

This paper details the successful development and early assessment of a novel mobile phone app designed to provide food-based information in an eating-out situation in a personalized manner.

## Introduction

### Eating Out

Eating out (defined as eating food that is prepared by others and consumed out of the home in food establishments such as restaurants, cafes, canteens, and fast food outlets) is a growing trend [1-3]. Food consumed out of the home can represent a significant contribution to daily energy intake [4,5] and is often higher in calories, fat, and sugar, lower in fiber, and served in larger portions than food consumed in the home [4,5]. It has been suggested that both the increasing practice of eating out and the increasing energy provision in this situation contribute to the obesogenic environment [1,2,6-8], and positive associations between the increased prevalence of eating out and high body weight in recent years have been made [6,8]. Obesity and its related conditions constitute a major public health concern, and strategies for prevention are required [9]. Furthermore, most adults underestimate their calorie intake when eating out and are therefore unlikely to mitigate their own risk [1,2].

### Food-Based Information

With an aim of reducing overeating, a regulation within Europe (EU No 1169/2011) makes it mandatory for all prepacked foods to display energy value and amounts of fat, saturates, carbohydrates, protein, sugars, and salt per 100 g or 100 ml food item [10]. Presently, only the United States has passed legislation requiring the provision of this type of labeling for out-of-home food provision [11], although voluntary labeling, particularly of energy content, is undertaken in various countries across the world [12]. Importantly, however, consumers are also asking for increased food-based information in the eating-out scenario [13-17], and the provision of information has been found to facilitate the adoption of healthier nutrition practices in this scenario [1-3,15,16,18,19]. Bates et al [1] and Burton et al [2] report lower repurchase intentions for unhealthy dishes following exposure to objective calorie and nutrient information. VanEpps et al [19] report fewer calories ordered following the provision of nutrition information, and Hammond et al [18] report fewer calories ordered and consumed following a nutrition-labeling intervention. Not all studies have reported benefits [20], but effects are typically small, and a lack of effects has been largely attributed to poor study size or methodology [20].

Food-based information provision in an eating-out situation, however, can result in menus or information boards that are described as cluttered and untidy, the consequences of which are that consumers feel overwhelmed and report that they would not use the information provided [13,16,17,21]. Consumers can report an unwillingness to search for information or high costs of this practice compared with benefits [1,22,23]. Food operators also acknowledge constraints to providing ingredient information on menus [15,16,21], including a reluctance to overload the menu with visual clutter, as well as a lack of knowledge on the part of the operator and a high perceived cost to the business, particularly where menus and dish specifications may change frequently [15,16,21].

Furthermore, different consumers can prefer or typically act on different types of information [2,16,17,22,24]. Yepes [24], for example, found menu calorie labeling was most valued by health conscious and older consumers and was effective in reducing calorie consumption in these individuals, whereas Burton et al [2] demonstrate impacts of calorie information on *low-health consciousness* consumers and impacts of calorie information and a color coding system for both *low-* and *high-health consciousness* consumers. Ellison et al [3] also found increased impacts of calorie information and a color-coding system compared with calorie-based information alone. The lack of effects of food information in some studies has been attributed to the type of information provided combined with the abilities of the consumer [20,25]. Different types of information may also be differentially effective in different settings. Bleich et al [20] and Dumanovsky et al [25] both report increased impacts of calorie information in fast food restaurants compared with sit-down type establishments. Food-based decisions are often made using simple heuristic processing methods [25], and decisions that are required under time pressure and high cognitive load can further be reliant on simple or automatic processing strategies, such as a calorie limit. Decisions that take place in a more relaxed setting, however, such as at home, can be more consciously taken and so more informed, for example, through the consideration of complex ingredient information [19,25].

### Food-Based Information Provision

For decades, printed mediums have been the most common platform used for information provision in an eating-out scenario, but digital or electronic menus can deliver rich knowledge in an efficient manner [16,26] and may offer a neat solution to the existing constraints highlighted earlier [16,22,26]. Several studies have highlighted the potential that technology may hold in providing information [16,22], and a small number of mobile phone apps have been developed specifically for providing food-based information [27,28]. *SmartAPPetite* encourages people to eat local and healthy food [29]. *Tapingo* provides university students with canteen-based information and allows consumers to order food [30], and the *Smartmenu system* discussed by Pieskä et al [31] allows consumers to browse a menu, check additional information such as nutrient profile, and order items. The potential value of mobile phone apps for providing information is also increased by the rapidly growing numbers of mobile phones. Penetration rates of 68.4% in North America and 64.7% in Western Europe have been reported, with estimations of use by 32% of the global population [32], ranging recently in the top 50 markets between 11.2% in Ethiopia and 82.2% in the United Kingdom [33].

Increased information provision via digital platforms may also enable transparency and evidence of greater integrity for the food service operator [16,17,26,34]. Consumers with specific dietary needs are often limited in their choices not just by their personal constraints but also by a lack of information available from serving staff or a lack of trust in the information provided [22,26,35]. Lack of control, insufficient knowledge, and a lack of trust in any information provided can be key concerns when eating out [22,23,26,34,36]. Trust is an important component of health-based decision making [34-36], and catering operators that are open and transparent demonstrate commitment and trustworthiness to consumers [17,34]. Furthermore, even if the actual content is not always used, consumers can be reassured by the presence of such information [37,38]. Thus, food operators will also potentially benefit from increased information provision [16,21,26].

To enable healthy decision making in an eating-out situation, communication with consumers is clearly required, but any such communication should be carefully considered to ensure that it is well understood, suitable for each consumer, and suited to specific dishes and food operators [1-3,16,17,22,24,28]. Many existing apps use generic recipe data and so provide only generic information [27,28,39]. This generic information typically provides only estimations of nutrient content and is rarely sufficiently detailed to protect consumers with allergies or other very specific dietary requirements. For increased specificity, information should ideally be provided for every specific recipe from every specific manufacturer or food operator. For many consumers, more detailed and specific information is preferable, and more detailed information could increase trust and return business for food providers. For allergens, very specific information is required.

### A Mobile Phone–Based Technological Solution

This study aimed to develop a mobile phone app to provide consumers with food-based information in an eating-out situation. The app was intended to provide information as required for current European legislation and as recommended by a number of public health bodies, also with the consideration of consumer desires. The study was undertaken for the workplace canteen situation. There is a growing acceptance that the food provided in a workplace canteen setting can have a significant impact on health [40], as this is a captive environment where the contribution of the meal served could constitute an important element of the overall diet owing to the frequency of use. It is estimated that most employees eat one or more meals per day while they are at work [41]. Promotion of healthy diets in the workplace will also have benefits not only for the individual but also for employers and society [40]. The workplace canteen also typically offers limited dishes that can be more easily, accurately prespecified than may be the case for a chef-led restaurant [21]. The study was undertaken in Europe with a focus on European consumers, but evaluations also extended the study beyond Europe.

## Methods

### Overview

The study was undertaken in 3 stages. First, the requirements for the app were elicited through consideration of current legislative and scientific literature and consultation with potential end users, and then prioritized with potential stakeholders using the Must have, Should have, Could have, Won’t have (MoSCoW) method [42]. Second, a mobile phone app was designed and developed using an agile approach. Finally, the developed app was evaluated at 8 public engagement events using the System Usability Scale (SUS) questionnaire [43,44] and qualitative feedback. Full ethical approval was granted from Bournemouth University Research Ethics Committee before commencement. The research complied with Directive 95/46/EC of the European Parliament on the Protection of Individuals and with Directive 2002/58/EC of the European Parliament concerning the processing of personal data and the protection of privacy.

### Stage 1: Defining the App Requirements

#### Eliciting Government Requirements and Recommendations

Current legislation and recommendations for Europe were obtained from the European Union and from relevant public health agencies in Europe.

#### Eliciting Consumer Requirements

Consumer requirements were gained from a search of the scientific literature and from consultations with consumers. The search of the scientific literature was undertaken using known articles and snowballing from these by also looking at cited and citing articles. Consumer consultations were undertaken using one mixed methods study comprising a qualitative and then quantitative component [45,46], and one qualitative study [47]. A formal review of existing apps was not undertaken as part of the development work. No comparable apps with the necessary specificity and flexibility of dish information existed as far as we were aware, and few apps are developed following (reported) formal consultation at an early stage with end users; thus, a review of existing apps was unlikely to be informative.

The consumer consultation work has been published in detail elsewhere [45-47]. Briefly, the mixed methods study [45,46] used 8 focus groups of canteen users (N=40), 2 groups in each of the 4 European countries (Denmark, France, Greece, and the United Kingdom), to elicit the criteria used for making food-based decisions in a canteen scenario and known formats of food-based information provision. The decision-related criteria were then used in a best-worst scaling questionnaire to ascertain the relative importance of these criteria for making food-based decisions in 452 employees (Denmark [N=100], France [N=100], Greece [N=100], the United Kingdom [N=152]), who had access to a canteen at their place of work. The full sample was largely composed of females (61.1%, 276/452), aged 20 to 29 years (51.3%, 232/452), who had completed some form of higher tertiary education (74.1%, 335/452), and who now worked full time (60.4%, 273/452) in occupations classified as technicians and associate professionals (74.1%, 335/452). The known formats of and preferences for food information were also considered in a second best-worst scaling questionnaire administered to the same individuals to ascertain the most preferred format for the provision of food information. The study was undertaken in several European countries to enhance the generalizability of the work. Focus groups were used in a small sample to ascertain relevant criteria of canteen use and consumer preferences for food information provision in depth, given a lack of existing data in this area. The questionnaire was then used to determine the relative importance of these criteria and information formats in a much larger sample. The best-worst scaling method requires respondents to choose their most preferred and least preferred option of several sets, allowing relative assessments of the criteria of interest without the use of absolute judgments that can differ between contexts and cultures.

The additional qualitative study [47] used 4 focus groups of canteen users from the United Kingdom (N=28) to confirm the information desired for food-based decisions in a canteen scenario and ascertain attitudes and opinions toward the use of information communication technology (ICT) in this context. Focus groups were again used in a small sample to ascertain attitudes and opinions in depth, given a lack of existing data in this area.

#### Prioritizing Requirements

Elicited requirements for the app were then prioritized using MoSCoW principles following full consideration by the research team and potential stakeholders to ensure wide use of the app and increased transferability. The MoSCoW method [42] is a technique used in software development to prioritize the importance of the delivery of all identified requirements. Requirements are categorized as *Must have*, *Should have*, *Could have*, and *Won’t have*, based on importance, and then prioritized during the development process in this order. Requirements identified as *must have* are considered central to project success; those identified as *should have* are considered important, but not necessary; those identified as *could have* are considered desirable but not necessary; and those identified as *won’t have* are considered least important [42]. The research team included academic researchers in eating behaviors, hospitality, and food service (KMA, JB, SP, FJAPC, and HH), academic researchers and developers in computing and app development (GL, NJ, and IM), caterers, food operators, and personnel from the catering industry (RC and MR) and researchers working within the food industry (LS and AG). Additional potential stakeholders from the food industry (caterers, food operators, and personnel from the food and hospitality industries) were also consulted. Each of the elicited requirements were discussed and considered for inclusion in the app.

### Stage 2: Designing and Developing the App

The app was intended for use in a workplace canteen using a predetermined food menu offering a number of dishes and side dishes per day and was envisaged as a consumer-facing user interface attached to a back-end database. All aspects of the app from the user’s perspective were carefully considered from visual esthetics of the user interface (eg, logo, color scheme, and picture placement) to the workflow of user tasks. A user journey map was first created to visualize the timeline of interactions with the potential app from the landing page. Wireframes of each app screen were then produced using Balsamiq (Balsamiq Studios). These wireframes focused on app screen layout and content structure and were organized to reflect the user journey map. These wireframes were then mapped to mockups showing the actual visual designs for each screen. An interactive prototype was created using InVision (InVision Technologies, Inc), and from this an Android app was developed using native Android Studio. Particular consideration was also required for the setup of the back-end database, as databases rely on precise definitions of entities stored, whereas natural languages do not have this level of precision and contain synonyms and terminology imprecision. For instance, the terms *dish*, *food item*, and *meal* needed clarification. The specification of ingredients, for example, milk, within ingredients, for example, cheese, within dishes, also added complexity.

The app was developed for Android mobile phones following Google’s Material Design Guidelines and industrial best practices, with reference to the adapted Technology Acceptance Model (TAM) [48-50]. The adapted TAM proposes that technology usage is positively predicted by *perceived usefulness* (“the degree to which a person believes that using a particular system would enhance his or her job performance”) [48], *perceived ease of use* (“the degree to which a person believes that using a particular system would be free of effort”) [48], *perceived enjoyment* (“the extent to which the activity of using the [technology] is perceived to be enjoyable in its own right, apart from any performance consequences that may be anticipated”) [49], and *perceived visual attractiveness* (“the degree to which a person believes that the [technology] is aesthetically pleasing to the eye”) [50].

Throughout app development, several consultation meetings were held with all stakeholders to make sure the app would meet all demands and support further needs. A prototype app was developed to include all requirements identified as *must have* and *should have* using the MoSCoW framework. This prototype was then assessed and refined by the research team to create a second version.

### Stage 3: Initial Evaluation of the App

Following development in 2016, version 2 of the prototype app was demonstrated to potential stakeholders and end users at several public engagement events in 2017, where assessments of the app were made using a questionnaire.

#### Public Engagement Events

Data were collected from adult participants at 8 global public research engagement events. These public engagement events were largely intended to promote research for the general public and included displays from a range of disciplines—that is, they were not held specifically to demonstrate or promote our app. The app was demonstrated, and data were collected at 4 public engagement events held in the United Kingdom, 1 event held in France, 1 event held in Denmark, 1 event held in Malaysia, and 1 event held in China. These events were chosen to represent the European consumers in the countries involved in the study, but also expanded consideration of the app to different markets. Demonstration at these events was intended to gain feedback and insights from a wide range of potential users of the app. These events are typically attended by members of the public who are educated and have an interest in science, education, and/or progress and growth. All attendees who were interested were permitted to try the app and provide data. There were no inclusion/exclusion criteria, to increase generalizability, but the information on the app was provided in English. Mock data on 4 mock dishes from 1 mock canteen were included in the app for demonstration purposes. Respondents were asked to view and manipulate the app for as long as they wished and then complete the evaluation questionnaire in paper form.

#### Evaluation

Assessments of the app were made using the SUS questionnaire [43,44]. The SUS consists of a 10-item questionnaire based on 5-point Likert scales (5=strongly agree to 1=strongly disagree) to assess usability. In total, 5 questions are positively phrased: “I think that I would like to use this system frequently,” “I thought the system was easy to use,” “I found the various functions in this system were well integrated,” “I would imagine that most people would learn to use this system very quickly,” and “I felt very confident using the system.” A total of 5 questions are negatively phrased: “I found the system unnecessarily complex,” “I think that I would need the support of a technical person to be able to use this system,” “I thought there was too much inconsistency in this system,” “I found the system very cumbersome to use,” and “I needed to learn a lot of things before I could get going with this system.” An additional question was also added to the end of the questionnaire “I believe the FoodSmart App will be useful to customers in a canteen setting to help them to get informed about the dishes offered.” There was also the opportunity for respondents to leave open-ended comments if desired. The SUS is recommended to assess usability, because it is technology-independent, short (and therefore easy to complete and analyze), and can provide a single score per person [51]. It was chosen for this study because it has been extensively used on a variety of products and systems, notably for measuring the functionality of apps. It is a well-validated instrument and supported by a large pool of comparison data.

#### Analysis

Questionnaire responses were entered into SPSS version 24. All questionnaires providing complete SUS data were used for analysis, although not all questionnaires included complete demographic data. The results of the tests for normality were acceptable, and the results of a confirmatory principal component analysis (Varimax rotation) on the responses from the 10 SUS questions were comparable with those conducted by other researchers [51,52]. Analysis of the raw item data gave 2 factors that explained 53.8% of the variance, consisting of (1) the 5 positively phrased items (explaining 41.4% of the variance) and (2) the 5 negatively phrased items (explaining a further 12.4% of the variance). Overall SUS scores based on the 10 SUS questions were calculated as described by Brooke [43]. Values of positively phrased items were reduced to a 0 to 4 scale by subtracting 1. Values of the negatively phrased items were subtracted from 5 to reverse their sense and to similarly reduce them to a 0 to 4 scale. The sums of the 10 items together were multiplied by 2.5 to provide individual SUS scores out of 100. SUS scores were analyzed using descriptive statistics and *t* tests. For significance testing, probability was considered at *P*<.05. The additional question given at the end of the questionnaire was considered as a separate individual question and analyzed using descriptive statistics only. Open-ended feedback comments were analyzed through open coding techniques and grouped into themes to provide an added richness of understanding.

## Results

### Stage 1: Defining the App Requirements

#### Government Requirements and Recommendations

European legislation requires the provision of information for all dishes served in an eating-out situation on 14 common allergens (celery, cereals, crustaceans, eggs, fish, lupin, milk, molluscs, mustard, nuts, peanuts, sesame, soybeans, and sulfur; EU regulation No 1169/2011). Recommendations at the European level also suggest the inclusion of information on calorie content and amounts of fat, saturates, carbohydrates, protein, sugars, and salt of a food per 100 g or 100 ml or per weight of portion served [10].

#### Consumer Requirements

The study by Price et al [45,46] revealed 8 criteria for food selection in a canteen setting: *Animal Welfare* (how an animal is coping with the conditions in which it lives, where an animal is in a good state of welfare if it is healthy, comfortable, well nourished, safe, able to express innate behavior, and is not suffering from unpleasant states); *Environmental Impact* (the effect the food production has on the environment); *Fair Trade* (Fair Trade aims to help producers to get a fair price for their products so as to reduce poverty, provide the ethical treatment of workers and farmers, and promote environmentally friendly and sustainable practices); *Naturalness* (the extent to which fresh ingredients are used, or that there is less use of processed foods containing additives and preservatives); *Nutrition* (the nutritional composition of the food); *Organic* (food produced in a way that respects natural life cycles, minimizes the human impact on the environment, and operates as naturally as possible); *Provenance* (where the food was grown/produced); and *Value for Money* (the ratio between the perceived quality of the dish and the price paid for it). Consumers recognized a variety of formats currently used to provide food-based information to consumers and expressed a desire for information that was easy to understand and easy to use, and preferences for only the information that was relevant to themselves, such as for their religious beliefs and their dietary preferences, eg, vegetarianism. Results from the questionnaire study on food-based decision making suggested that in all 4 European countries, the criteria of Nutrition, Naturalness, and Value for Money were those most valued by consumers, followed by criteria based on Animal Welfare, Organic foods, and Provenance, followed by those of Environmental Impact and Fair Trade [45]. Results from the questionnaire study on food-based information provision suggested that in all 4 European countries, the formats most preferred by consumers were traffic light labeling, information boxes, and quality assurance markings. Latent class cluster models also identified 5 clusters of consumers in relation to information use, described as *Heuristic Processors* (individuals who preferred easy-to-find-and-use information); *Brand Orientated* (individuals who were persuaded by brand authority); *Systematic Processors* (individuals who prefer more detailed information); *Independent Processors* (individuals who use a mix of heuristic and systematic processing); and *Tech-Savvy* (individuals who indicated a high preference for technology and interactive displays) [46]. Some differences between countries were also found [45,46].

The study by Bray et al [47] confirmed the importance for consumers of information on nutritional content (Nutrition), ingredients and allergens (Naturalness), and confirmed desires for information to be presented in a clear and simple manner, desires for different information by different consumers, and a desire to personalize the information that each person receives. Options to remove or override any personal preferences were also preferred, as opposed to limits to free choice. In relation to ICT, consumers currently used ICT for food-based reasons related to: *Marketing*, for example, discounts or loyalty schemes; *Increased convenience*, for example, Web-based booking and viewing menus in advance; and *Accessing additional information*, for example, identifying ingredients and customer reviews. Avenues for future ICT usage focused on the following: the provision of digital menus and increased information per dish; the consumer gaining control over and confidence in what they were eating; the value of personalized (relevant) information provision; and the value of additional information/free choice despite personalization. The idea of using a mobile phone app to provide personalized information was generally supported.

The literature in the field was also found to highlight similar opinions and concerns regarding food-based decision making [13,21,38,53] and supports similar conclusions regarding the provision of nutritional information in a retail setting [15,16,37] and in an eating-out setting [3,19,38,53]. Similar opinions toward technological solutions were also found [15,16,53].

#### Requirement Priorities

The priorities for the app based on MoSCoW principles [42] are presented in Table 1.

**Table 1:** MoSCoW (Must have, Should have, Could have, Won’t have) requirements for the app.

MoSCoW	Requirements^a^
Must have	Provide detailed and accurate dish information as supplied by the manufacturer, including ingredients and allergens; Include nutrient information (calories, sugar, fat, saturated fat, and salt); Include information allowing dietary classifications; Include price per dish, allowing assessments of “value for money”; Provide the information in an easily accessible format; Enable quick information access, eg, via a QR (quick response) code; Allow users to store personal preferences about dietary needs and requirements, for example, religion, vegetarian, and vegan; Tailor menu presentation based on user profile; Warn users for certain dishes based on user preferences, for example, allergens and religious dietary needs
Should have	Adopt a traffic light type coding system for the nutritional information; Provide additional detailed information if required; Provide a calorie calculator allowing assessment of a whole meal composed of several dishes; Allow users to set a desired calorie limit per dish; Allow presentation of all dishes to retain free choice for the consumer while retaining a tailored presentation based on the user profile
Could have	Provide information about ingredient provenance and organic nature; Provide information about animal welfare, environmental impact, and fair trade nature of all ingredients; Allow users to set favorite food region; Allow users to set favorite dish or specific food items; Enable recommendations based on user preferences; Store previous purchase history; Enable recommendations based on previous consumption; Provide warnings of over or excess consumption; Provide personalized food messages for each user; Allow sharing via social media; Allow users to take photos of dishes/meals chosen; Allow users to search for dishes; Allow users to access menus in advance; Include functionality to preorder meals; Include functionality to feedback dish choices to a canteen; Include functionality to feedback comments/suggestions to a canteen;
Won’t have	Provide generic dish information; Limit consumer choice; Provide information on allergen traces; Provide advertisements; Support push notifications, for example, for special offers; Include functionality to allow users to pay via the app; Include functionality to feedback sales to a canteen

^a^Definitions: a) Dish: can be made up of several food items, for example, lasagna with side salad; b) Food item: something a consumer can buy, which has nutritional facts and can fit a food classification; c) Nutritional fact: a fact about the nutritional values of a food item (eg, salt level or sugar level); d) Food classification: information about food items in relation to dietary classifications such as vegetarian, vegan, kosher, or halal.

### Stage 2: Designing and Developing the App

The app was developed as a consumer-facing user interface attached to a back-end canteen-based database. The back-end database held all required information per dish (ingredients, allergens, and nutritional composition), as supplied by caterers and food manufacturers. The app was designed such that caterers and food manufacturers would be given free and unlimited access to the database to upload information for as many dishes as they wished based on their own dish specifications and could update this information as often as they wished. Information in the database is stored per canteen.

The user interface was designed to allow consumers to view all information provided by caterers and food manufacturers, and to manipulate the information displayed if desired, through the selection of settings on the user interface that allowed consumers to input personal details and preferences. The information for each dish could be revealed by accessing a menu or by scanning a QR (quick response) code placed on a menu or dish label, thus allowing very quick access to all information if desired.

Particular consideration, throughout development, was given to the storage and security of personal data from users and dish and ingredient data from manufacturers; thus, all personal information is stored on the user’s device, and all industry data are owned and managed by the operator.

Version 1 of the prototype included features to meet all requirements identified as *must have* and most of the features identified as *should have* using the MoSCoW method [42]. Full details of the user interface are given in Table 2. In addition to the features identified, version 1 of the app also included functionality for users to label dishes that they liked, but this functionality has not yet been linked to databases to allow feedback to caterers. Version 2 of the prototype app included all features identified as *must have* and *should have*, and so included all features of version 1, plus 3 additional features.

**Table 2:** Details of the prototype app per user interface screen.

Screen		Display	User actions
**Basic (nonpersonalized) functionality**			
	Screen 1	Welcome and option for tutorial	User swipes to progress
	Screen 2	Personalization screen	User has option to personalize the app (personalized functionality) or skip this (basic functionality)
	Screen 3	Display of local canteens with available information based on Global Positioning System locator	User selects desired canteen and clicks option to see menu
	Screen 4	Menu for the day is displayed pictorially, consisting of dish name, picture, price, and diet classification	Users can view all dishes available. Users can view full information per dish by clicking on any dish
	Screen 5	Information (description, energy, portion size, ingredients; allergens; and nutritional content (gram per 100 g) of fat, saturated fat, carbohydrate, sugars, fiber, protein, salt, using the traffic light system) is displayed for the dish	User can view all information. User can also toggle a heart symbol to send feedback to the caterer that they like the dish. Activation with the QR (quick response) code (per dish) results in immediate arrival at Screen 5
**Personalized functionality (optional)**			
	Screen 2	Personal preferences are available based on the following: Canteen selection (local canteens available); Diet type: vegetarian, vegan, pescatarian, halal, kosher; Allergens: celery, cereals, crustaceans, eggs, fish, lupin, milk, molluscs, mustard, nuts, peanuts, sesame, soybeans, sulfur; Dish calories	User selects preferences for canteen, diet type and allergens by moving a bar from “selected” to “not selected.” These are saved automatically on the consumer’s mobile phone and remain stored or can be updated at any time. The default selection is “not selected.” Users can also set a desired maximum amount of calories per dish using a sliding scale and is provided with WHO (World Health Organization) recommendations for men and women
	Screen 4	When personal preferences have been selected, the menu is provided such that preferred dishes are provided at the top, and less preferred dishes are provided at the bottom of the list. Dishes that do not fit the user profile based on diet type and allergens are provided grayed over	Users can view all dishes available. Users can view all information per dish by swiping across the dish

First, the information provided per dish was no longer presented on a single screen but split over 3 screens (overview/description; nutritional information; and ingredients/allergens) to facilitate users to access only desired information and avoid information overload. Second, the app included a calorie calculator. This facility allowed consumers to select the dishes they intended to consume, and a value for total calorie content was automatically calculated and provided for the meal as a whole. This facility recognizes that individuals do not typically only consume single dishes. Finally, the app included a notification system allowing caterers to send messages to users, for example, on special offers and promotions, recommendations, or advice. This feature has also not yet been fully activated.

**Figure 1:**
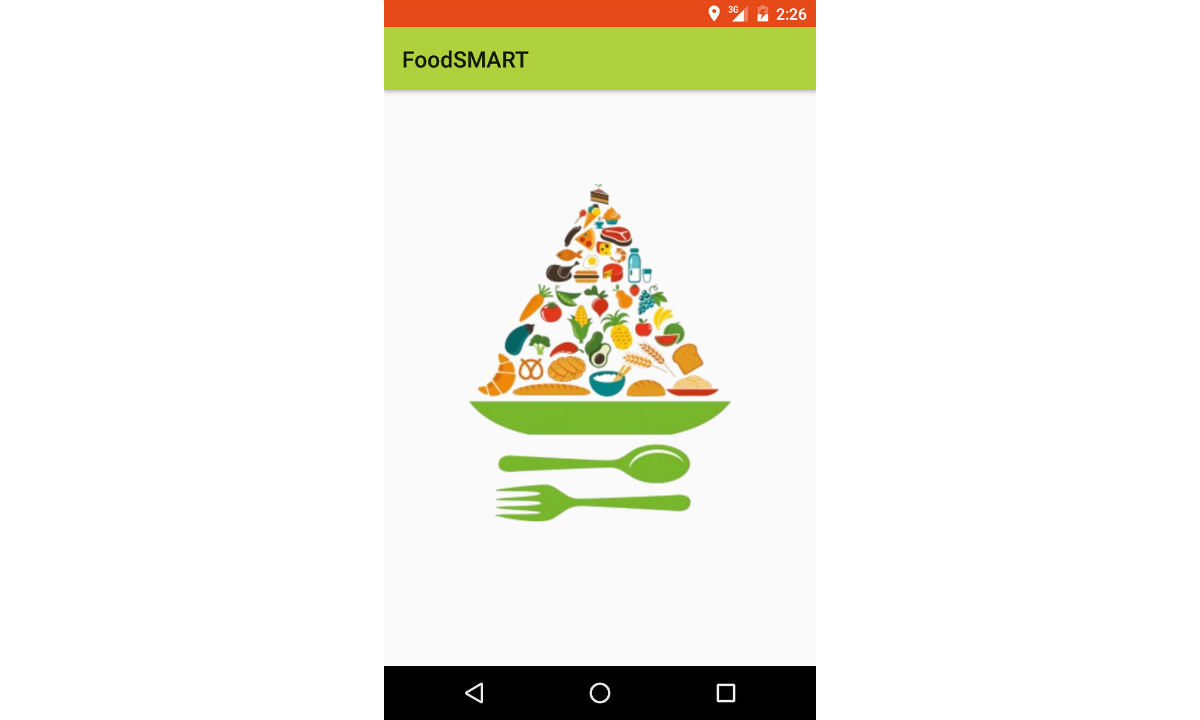
Screenshot of the FoodSMART app: screen 1.

**Figure 2:**
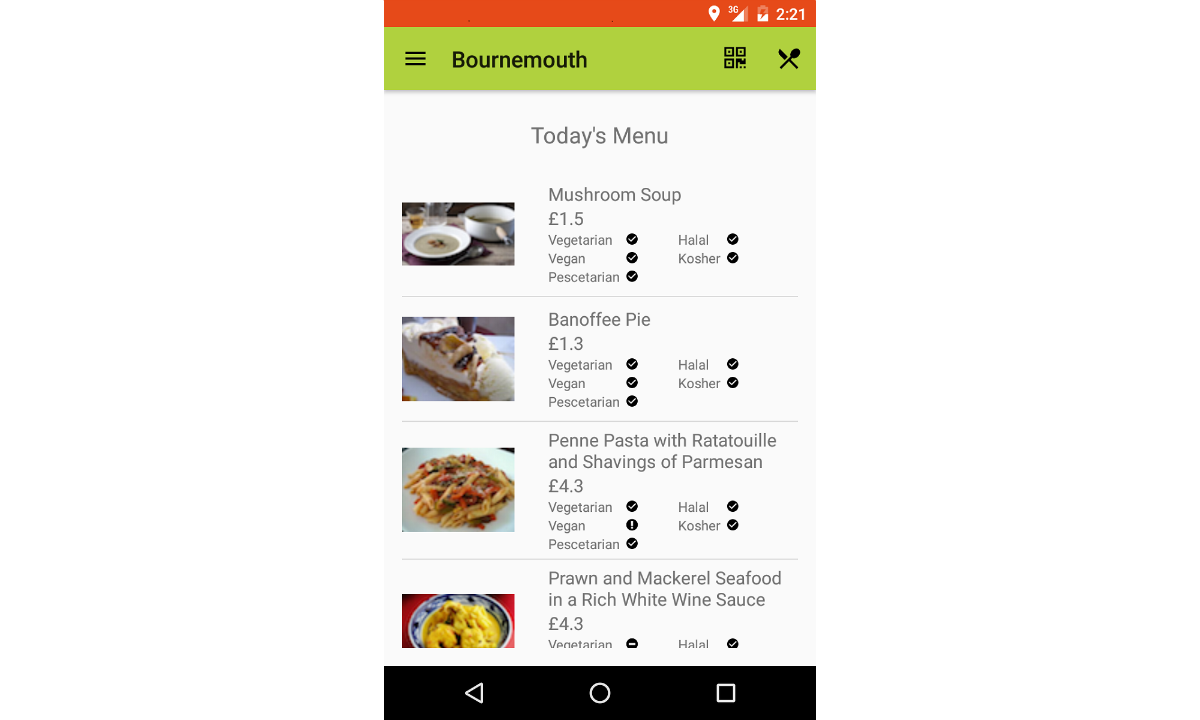
Screenshots of the FoodSMART app: screen 4.

**Figure 3:**
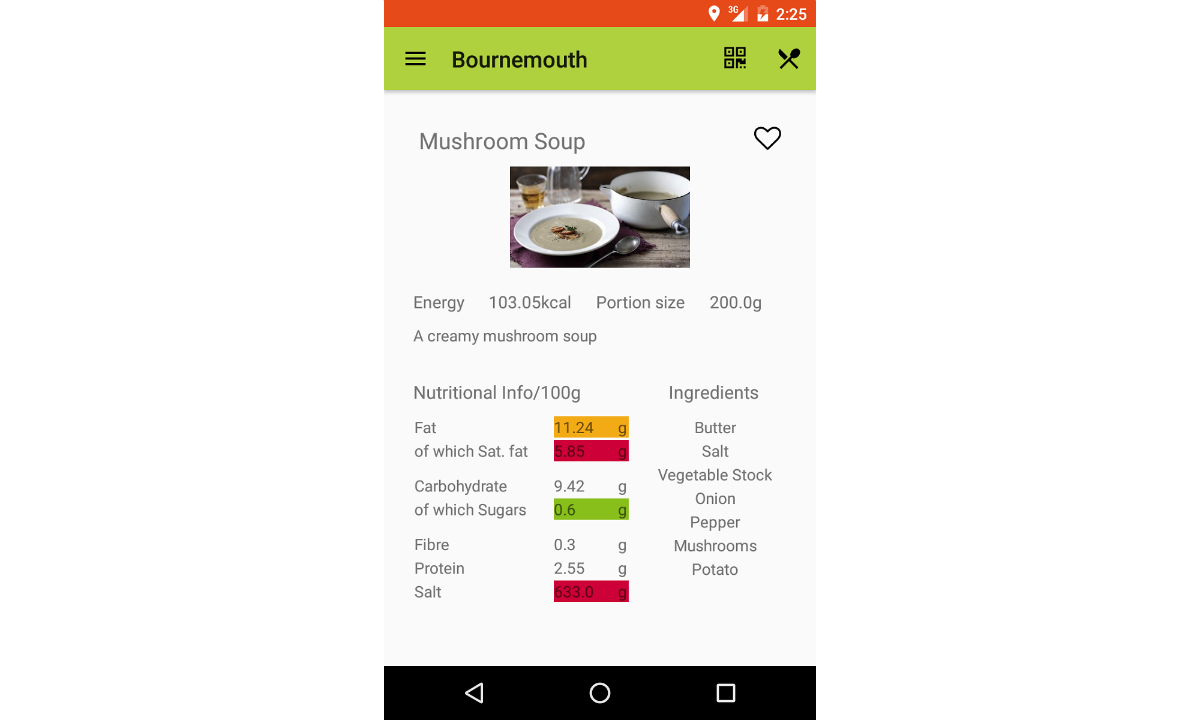
Screenshots of the FoodSMART app: screen 5.

Details of the app, per screen, are given in Table 2. Images of the app screens are also displayed in Figures 1-3. The app is currently available for download from the Google Play Store. A video demonstration of both versions of the prototype app can be viewed in the dissemination section of the project website [54].

### Stage 3: Initial Evaluation of the App

A total of 233 respondents evaluated the prototype app and provided usable questionnaire data: 79 respondents from the UK events, 54 respondents from the French event, 18 respondents from the Danish event, 34 respondents from the Malaysian event, and 48 respondents from the Chinese event. Of these, 81 (34.7%, 81/233) were male, 143 (61.3%, 143/233) were female; 115 (49.3%, 115/233) were aged 20 to 29 years, 39 (16.7%, 39/233) were aged 30 to 39 years, 35 (15.0%, 35/233) were aged 40 to 49 years and 32 (13.7%, 32/233) were aged 50 years and older. Demographic details were incomplete from some respondents.

The mean overall SUS score was 68.3 (SD 15.4). Responses to individual questions are given in Table 3. Mean responses for all questions were positive relative to the midpoint of the scale and so represented a positive perception of the app (smallest *t*_229_=26.40; *P*<.01).

Total SUS scores by gender, age, and event location are given in Table 4. Scores were comparable for males and females and no linear trend with age was found (largest *t*_196_=0.81; *P*=.42). Differences were found between event locations, where higher total SUS scores were found in the United Kingdom and Denmark, compared with France and also with Malaysia and China (*F*_4,201_=11.96; *P*<.01).

The following common themes emerged from the open-ended feedback:

- Good for informing healthy choices: Consumers liked the provision of various health and nutritional information in a manner that they found useful, for example, “Very useful for health conscious people, especially those who are busy, usually hard for them to buy healthy foods they wanted.”
- Particularly useful for those on restricted diets: Some participants highlighted a particular interest for those consumers who are health conscious or observing a restricted diet, for example, “Extremely useful and important app that would also be important to people with dietary restrictions.”
- Easy to use: Consumers offered support for the provision of information in a simple manner, and the use of the QR code, menus, and screen swipes to aid full use.
- Support for the personalization aspect and specific functions: The concept of using a mobile phone app to access personalized information on the nutrients, ingredients, and allergens was viewed positively by many participants. The calculator function was well received, and respondents felt it was easy to use, for example, “Very interesting and promising tool for personal use as well as for service providers—the calculator function is good.”
- Concerns around data accuracy: A small number of respondents expressed concerns around the possible accuracy of information provided through the app, not realizing the direct link between the caterer or food manufacturer and the information provided.
- A wish for improved functionality: Some consumers also suggested a desire for additional features, such as links with social media, or an option to provide feedback to suppliers about missing or questionable information.

**Table 3:** Mean and standard deviation of responses to all individual questions in the SUS questionnaire plus the additional question, for all consumers (N=233).

Question	Value, mean (SD)^a^
I think that I would like to use this system frequently	2.4 (1.1)
I found the system (was not) unnecessarily complex	2.8 (1.0)
I thought the system was easy to use	2.8 (0.9)
I think that I would (not) need the support of a technical person to be able to use this system	3.0 (1.1)
I found the various functions in this system were well integrated	2.7 (0.8)
I thought there was (not) too much inconsistency in this system	2.7 (0.9)
I would imagine that most people would learn to use this system very quickly	2.9 (1.0)
I found the system (not) very cumbersome to use	2.7 (1.0)
I felt very confident using the system	2.6 (1.0)
I did (not) need to learn a lot of things before I could get going with this system	2.7 (1.1)
I believe the FoodSmart App will be useful to customers in a canteen setting to help them to get informed about the dishes offered	3.1 (0.9)

^a^0=strongly disagree and 4=strongly agree.

**Table 4:** Mean, SD, minimum, and maximum total System Usability Scale scores by gender, age, and event location for all consumers (N=233).

Demographic characteristic^a^		SUS^b^ score, mean (SD)	Minimum-maximum
**Gender**			
	Male (n=81)	69.8 (14.6)	32.5-100.0
	Female (n=143)	67.9 (15.8)	27.5-100.0
**Age (years)**			
	20-29 (n=115)	67.0 (13.6)	27.5-97.5
	30-39 (n=39)	73.9 (14.2)	35.0-97.5
	40-49 (n=35)	64.4 (19.7)	32.5-95.0
	50+ (n=32)	71.9 (16.4)	45.0-100.0
**Event location**			
	United Kingdom (n=65)	76.3 (13.7)	42.5-100.0
	France (n=50)	67.8 (14.4)	32.5-95.0
	Denmark (n=15)	73.0 (16.8)	45.0-97.5
	Malaysia (n=30)	62.8 (9.6)	35.0-75.0
	China (n=42)	58.6 (15.6)	27.5-87.5

^a^Numbers by gender, age, and event location do not equal 233 owing to incomplete demographic information in returned questionnaires from some respondents.

^b^System Usability Scale.

## Discussion

This study aimed to develop and evaluate a mobile phone app to provide consumers with food-based information in a workplace canteen setting in a manner that also allowed them to limit and/or personalize the information they received if desired. Initial stages of the study ascertained the information that European consumers (legally) should and would like to receive about the food on offer and established that consumers would like to personalize this information and receive this information in a certain manner. A mobile phone app was subsequently developed to address these identified needs. Early assessments of the developed app suggest support for the app and use, if available.

Consumers were able to provide clear suggestions for information provision, which largely matched those provided by current legislation and previous research. App development resulted in a fully functioning prototype app, and the prototype was then evaluated positively. The SUS feedback gained highlights the strong usability and clarity of the developed app. Our app received a mean SUS score of 68.3. According to Bangor et al [51], scores in the range of 68 to 71 are typical of customer premise equipment (eg, phones and modems), graphical user interfaces, and interactive voice response phone systems. According to Kortum and Bangor [55], a score of 68.3 is about the value for Global Positioning System systems and slightly lower than that for digital video recorders. Furthermore, qualitative comments reaffirmed the potential ease of use and emphasized the value of information that was provided in a simple manner. Comparable SUS scores were also found between males and females and regardless of age, suggesting comparable appeal, but the app was found to be more positively evaluated in the United Kingdom and Denmark, compared with France and then compared with Malaysia and China. These location-based differences may warrant further examination and highlight a need for cultural considerations in app development and testing.

The study has confirmed that many consumers seek additional information when eating out and are keen to consider the constituents and nature of the food that they eat [13,15,16,53]. This study also confirms that the nature of the desired information can vary between consumers [1,2,13,16] and that consumers like the idea of personalized information [13]. The use of mobile phone technology facilitated development and was considered appropriate by consumers. The use of mobile phone technology to display detailed information on menus has been demonstrated previously to be successful [16,38], with particular focus on increased capabilities to navigate quickly and the provision of added value information that is integrated fundamentally and placed prominently [16].

Other studies have also indicated that consumers have greater trust in information provided through a technological solution rather than that gained from serving staff [16,17], and although a small number of respondents expressed concerns around the possible accuracy of information provided through our app, it may be that some additional information is required to inform consumers of the close links between the app and food providers, and the abilities for immediate and frequent updates where necessary. Some studies also report a need for more traditional forms of information provision to supplement technological information provision [53], but increased familiarity with technological solutions may reduce these concerns.

Components of the app were also designed to benefit caterers and food providers. The app is quick and easy to populate and does not take technological skill or experience. Difficulties were encountered during the development stage, however, owing to competing requirements and considerations from different stakeholders. Competition over requirements was experienced among stakeholders dependent on their intended end-use of the app. Difficulties also arose as a result of the differential use and consideration of similar terms and similar concerns between stakeholders, and the relative importance given to issues such as consumer privacy and data storage.

Further testing of our app is required. The app has been demonstrated as likely to be used and potentially useful, but further research is required in real canteen settings, both in terms of consumer acceptability and in terms of value to the consumer. Work is required specifically to demonstrate the value of the app for improving meal choice in a canteen scenario, for example, through the greater selection of more healthy meals, and for improving consumption, for example, through the consumption of less daily calories. Various research demonstrates that increased information may not necessarily be used or does not benefit all consumers [15,25,53], and some research even demonstrates less healthy consumption following the provision of nutritional information [56]. Considerable research demonstrates a strong distinction between the intention to perform a behavior and actually undertaking that behavior [57]. Behavioral tests of the benefit of the app are required. Preferably, these tests would be conducted in the form of randomized controlled trials to reduce potential bias, using behavioral outcomes based on food selection and consumption, and where calculations of benefits for health compared with relative costs would also be possible. We also accept that not all canteen users would use a mobile phone app, thus testing needs to be conducted in an appropriate volunteer sample.

Evaluation of the app from a service provider’s perspective may also be of value. Consideration of additional criteria or additional functionality for food service operators such as feedback mechanisms could be of use. Food service providers are increasingly using digital means for informing and understanding consumers [15,16,58]. Some of the *could have* criteria that were not addressed during our development could be implemented. Facilities for consumer feedback, for example, via stores of purchases and purchase history may be beneficial.

Facilities for providing recommendations based on purchase storage and history may also be desirable for consumers. Using purchasing data from 39 burger restaurants, it has previously been found that software recommendations can change the mix of items purchased. The share of adult main course items requesting *no sauce* increased by 6.8%, the share of children’s meals with apples instead of chips rose by 7.0% and the share of a breakfast sandwich without sausage increased by 3.8% [59]. Although these changes indicate only modest order refinement, the results suggest that targeted, adaptive food-based information could have behavioral potential. Facilities for social comparison may also be beneficial. Many apps related to social activities include a *share* option to allow others to view choices or allow comparisons between users or with an established norm. Notably, our app also currently does little to foster motivation for changes from the consumer. Engagement with the app requires consumers to be motivated to gain food-based information and then to act on this, and additional functionality, for example, feedback options for others to comment on food choices, through *likes*, may encourage and facilitate this motivation and so facilitate engagement with and action based on the app. Increased information provision may also be desirable to include an ability to flag any ingredient as an allergen. Allergies exist to more than the specified 14 commonly occurring allergens, and consumers have previously expressed a desire for all ingredients to be considered as potential allergens [26].

Benefits for health may also be enhanced by the inclusion of additional features not requested by the consumer. Considerable interest is currently focusing on the use of *nudges* to encourage consumers to select and consume more healthy dishes, or those that are more sustainably sourced [60-62]. The order in which items are displayed on the app may *nudge* individuals to select dishes toward the top of the list compared with those further down [62]. Tests of strategies such as these would be of interest.

Additionally, different versions of the app may be desirable, for example, through the use of different formats, different controls, or different setups. Tsai and Cheng [63], for example, identified 4 consumer clusters based on willingness to use technology (technology explorers, technology recipients, technology optimists, and technology laggards), and the app may be differentially viewed by these groups. Others also report the different use and preference of technology in different consumers [16,59].

Consideration of the non-European market in testing and development would also be of value. Food provision and the interests and needs of consumers can differ widely between countries and cultures, and further consideration of these differences is required. Although our app was developed in Europe and may be transferable to other Western cultures, it may not transfer to other more developing cultures where food provision is less regulated, for example, where the majority of takeaway food is sold by individual street vendors.

In conclusion, this study demonstrates the development and early positive evaluation of a prototype mobile phone app for the provision of food-based information in a canteen scenario in a manner that can be personalized. The study also confirms an interest by consumers in food-based information provision in eating-out scenarios, demonstrates a wish by consumers for abilities to personalize and limit the information provided, and demonstrates the value of a mobile phone app as a potential solution to current needs. Further research allowing further refinement of the app and demonstrating a health benefit from use of the app is required.

## Abbreviations

ICT: information communication technology
MoSCoW: Must have, Should have, Could have, Won’t have
QR: quick response
SUS: System Usability Scale
